# Mapping Psychosocial Challenges, Mental Health Difficulties, and MHPSS Services for Unaccompanied Asylum-Seeking Children in Greece: Insights from Service Providers

**DOI:** 10.3390/children11121413

**Published:** 2024-11-23

**Authors:** Ioanna Giannopoulou, Gerasimos Papanastasatos, Eugenia Vathakou, Thalia Bellali, Konstantia Tselepi, Paraskevas Papadopoulos, Myrsini Kazakou, Danai Papadatou

**Affiliations:** 1 2nd Department of Psychiatry, Faculty of Medicine, School of Health Sciences, Attikon University General Hospital, National and Kapodistrian University of Athens, Rimini 1, 12462 Athens, Greece; 2 Department of Nursing, School of Health Sciences, National and Kapodistrian University of Athens, Papadiamantopoulou 123, 11527 Athens, Greece; gpapanastasatos@kethea.gr (G.P.); evathakou@hotmail.com (E.V.); dpap@nurs.uoa.gr (D.P.); 3 Department of Nursing, School of Health Sciences, International Hellenic University, Sindos, P.O. Box 141, 57400 Thessaloniki, Greece; thalia@ihu.gr; 4 Merimna—Society for the Care of Children and Families Facing Illness and Death, Frangon 13, 54626 Thessaloniki, Greece; tselepi@merimna.org.gr; 5 Information Technology Department of Sismanoglio General Hospital, General Hospital of Attica “Sismanoglio-Amalia Fleming”, Sismanogliou 1, 15126 Athens, Greece; pdesper@gmail.com; 6 UNICEF Greece, Agias Lavras 91, 15773 Athens, Greece; mkazakou@unicef.org

**Keywords:** unaccompanied refugee minors, asylum-seeking children and adolescents, mental health, perceived psychosocial needs, MHPSS services, mapping

## Abstract

Background/Objectives: Evidence-based information is crucial for policymakers and providers of mental health and psychosocial services (MHPSS) for unaccompanied asylum-seeking children (UASC). However, there is a scarcity of national-level studies investigating the MHPSS needs of UASC and how these are addressed in Greece. The research objectives of this study were to explore: (a) the psychosocial and mental health needs of UASC living in Greek long-term accommodation facilities as perceived by MHPSS providers, and (b) the range of services across the country, highlighting gaps and best practices in service delivery. Method: An exploratory, predominantly quantitative design was adopted to map UASC’s psychosocial difficulties, mental health problems, and MHPSS delivery. Purposive sampling was implemented, with 16 of 17 NGOs operating long-term accommodation facilities for UASC and 16 child and adolescent mental health services (CAMHS) participating. The sample included 79 participants (34 facility coordinators, 28 field psychologists, and 16 CAMHS directors). A 5-W mapping tool (Who, Where, What, When, and Which) was used for data collection, through an online survey. Data analysis involved quantitative and qualitative methods (content analysis). Results: Of 798 minors, almost 59% showed signs of behavioral or emotional disturbance, with over half referred for psychiatric assessment and 27.7% needing inpatient care. Aggression, disruptive behaviors, self-harm, and suicidal ideation were the most challenging issues. CAMHS directors reported a high rate of crisis-driven responses, with 42.1% of UASC needing emergency psychiatric evaluation. Psychosocial support was hindered by communication difficulties, lack of a shared care philosophy, understaffing, job insecurity, and limited resources. Conclusions: Our findings highlight the mental health needs of UASC, and the challenges faced by facility coordinators, psychologists, and community mental health specialists. Future research should focus on the institutional and organizational factors influencing service delivery to improve support for UASC.

## 1. Introduction

In 2015 and 2016, Greece experienced a significant influx of refugees, becoming a major gateway country for asylum seekers heading to the EU. Due to the EU–Turkey Statement of Cooperation aiming at stopping the flow of irregular migration via Turkey to Europe, alongside stricter border controls in neighboring European countries [1], irregular migration became exceedingly difficult. As a result, thousands of refugees, including UASC were compelled to stay in Greece and apply for asylum [2,3]. Previously seen by most migrants as a temporary stopover, Greece hence became a long-term host country, tasked with providing a variety of services and assistance to them.

Unaccompanied and asylum-seeking children (UASC) are particularly vulnerable to developing mental health issues, due to the unique challenges they face. Numerous studies following the 2015 European migrant crisis have documented across national and settlement contexts high rates of mental health problems, such as post-traumatic stress symptoms (PTSS), post-traumatic stress disorder (PTSD), depression, anxiety, internalizing and externalizing behaviors, and somatic complaints among UASC [4], in keeping with previous research on refugee children resettled in high-income countries [5]. Trauma exposure, such as the number and severity of adverse events during premigration, the migration journey, and post-arrival to a host country is linked to mental health difficulties [5,6,7,8]. Depression and anxiety remain persistently high among unaccompanied minors, often associated with discrimination, limited language proficiency, and daily stressors [4]. Persistence of poor mental health outcomes was related with placement type (particularly in detention centers) and referral problems to mental health services [4], low levels of social support, and refusal of granting an asylum status [9].

Given the large number of unaccompanied minors’ arrivals in Greece, few studies and reports have provided critical insights into their situation in Greece. These studies highlighted the lack of a coherent framework for establishing and operating NGOs and services to support UASC, which has resulted in short-term and fragmented interventions by various NGOs, local authorities, municipalities, and civil society activists, creating a significant systemic problem [3]. Moreover, a study conducted by Digidiki and Bhabha [10] highlighted the absence of an integrated child protection system that spans national and local jurisdictions and identified several factors for the physical and psychological wellbeing of accompanied and unaccompanied minors: (a) an insufficient number of and a lack of properly trained and qualified staff to work with this uniquely vulnerable population; (b) risky living conditions inside camps; (c) potentially hazardous and unsupervised commingling of children with the adult migrant population; (d) a weak and insufficiently resourced child protection system; (e) a lack of coordination and cooperation among responsible actors; and (f) an inefficient and radically inadequate relocation scheme. Light was also shed upon the commercial sexual exploitation of unaccompanied minors, who rely on selling sex to raise money for their survival or to pay smugglers for their onward journeys [11].

The mapping study conducted by the Rosa Luxembourg Foundation regarding the national protective framework for unaccompanied minors, reformed in the first half of 2018 to address all identified shortcomings and gaps in the protection of refugee minors, highlighted the clear discrepancy between the legislative framework and the day-to-day practices. It exposed a fragmented child protection system, primarily due to the absence of an efficient guardianship system to navigate unaccompanied minors through complex reception and asylum procedures in Greece. Additionally, it pointed out the poor identification and reception conditions, as well as the lack of well-defined protocols and individualized care plans to address the psychosocial and mental health needs of UASC [12].

The rapid assessment of the mental and psychosocial needs of unaccompanied minors in Greece, commissioned by UNICEF in April 2017 and conducted through the Greek Institute of Child Health (May–July 2017), indicated a relatively low prevalence of severe psychiatric disorders requiring medication or hospitalization among unaccompanied minors in Greece, as compared to the rates of acute or chronic psychosocial conditions [13]. The report concluded that while expanding the availability of specialized services for this smaller group of UASC is critical, greater investment was required both in preventive measures and in early detection of UASC with mental health and psychosocial support (MHPSS) needs, including those suffering from internalized symptoms that are harder to detect, and those requiring treatment and support, in order to prevent chronic conditions that affect emotional wellbeing, social functioning, and cognitive and physical health [13].

In 2018, Greece enacted three laws—Law 4554/2018 [14], Law 4558/2018 [15], and Law 4540/2018 [16]—to address gaps in the protection system and enhance support for unaccompanied refugee minors. These laws established a comprehensive guardianship framework, improved the structure of guardianship services, and enhanced reception and identification procedures for asylum seekers. In this context, the path to safe long-term accommodation in shelters and supported independent living apartments (SILs) for minors older than 16 offered protection from precarious living conditions and provided them with access to legal advice, to school, to health services, and opportunities for social integration. In March 2020, two years after the publication of the Law 4540/2018, 1302 UASC were residing in shelters, 133 in SILs, and 3817 were awaiting placement [17]. In light of a notable scarcity of national-level studies that provide evidence-based information for policymakers and providers of MHPSS services for UASC in long-term accommodation facilities in Greece, the present mapping study was designed to address this gap.

The objectives of this research study were twofold: (a) to explore the psychosocial and mental health needs and challenges of UASC as perceived by MHPSS providers, and (b) to identify the range of MHPSS services available to UASC in long-term accommodation facilities across the country, highlighting gaps and best practices in service delivery.

## 2. Material and Methods

### 2.1. Procedure

First, a list of all non-governmental organizations (NGOs) operating long-term accommodation facilities in Greece in 2020, as well as all the facility coordinators, was obtained from the National Centre for Social Solidarity (EKKA). Second, permission to conduct the study was sought from the Board of Directors of 17 NGOs operating 53 long-term accommodation facilities at the time. A total of 16 out of 17 NGOs that operated 31 shelters and 3 SILs throughout the country consented to study participation. An email was sent to all facility coordinators, providing a brief overview of the study’s purpose and requesting contact information for psychologists employed in their respective residential structures. The psychologists needed to meet the prespecified inclusion criterion of having at least two years of experience in supporting UASC.

Data collection took place between March and October 2020 through an online survey. An email was sent out to 101 facility coordinators, field psychologists, and CAMHS directors with a written overview about the study, their ethical rights (e.g., voluntary participation and confidentiality of their responses), contact information for the research team for any questions, and a survey link. Reminder emails were sent after two and four weeks. Participants did not receive any compensation for their participation in this study.

### 2.2. Participants

**Figure 1:**
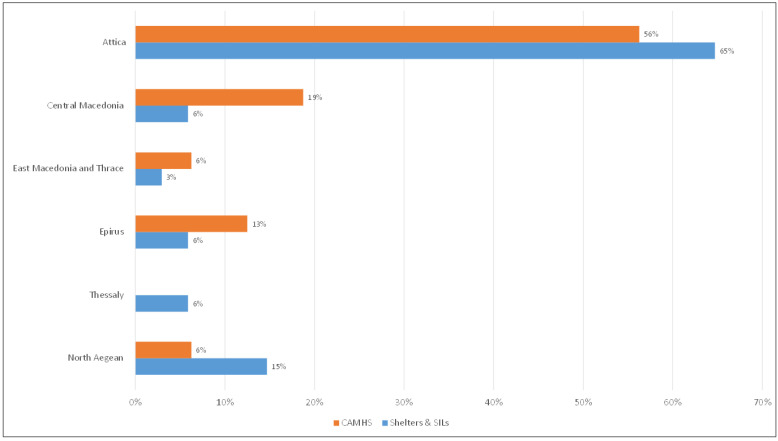
Shelters, SILs, and CAMHS by geographical location.

A total of 79 out of 101 participants who were invited to take part in the study responded to the survey. The final sample comprised 34 facility coordinators (responder rate 75.6%), 28 field psychologists (responder rate 70%), 15 directors of public community or hospital-based Child and Adolescent Mental Health Services (CAMHS), and one director of an NGO operating a specialized mental health service for migrants (responder rate 100%). Figure 1 depicts the distribution of shelters/SILs and CAMHS based on the geographical locations where the study participants came from.

### 2.3. The Survey

The questionnaire survey which aimed to answer the research objectives was developed following the conceptual framework of the 4-W mapping tool, designed by the Inter-Agency Standing Committee (IASC) Reference Group on Mental Health and Psychosocial Support in Emergency Setting and applied in various humanitarian settings in pre-emergency or emergency situations and refugee camps [18,19]. In the present study, the questionnaire included 5 mapping topics: Who is Where, When, doing What, and with Which resources or lack thereof (see Table 1). The questionnaire was then reviewed for its content by a child protection specialist at the UNICEF Greece Country Office (M.K.). While most questions were closed and provided a list of possible answers, this list was not intended to be exhaustive, and participants were given the choice to check “other” and provide their own response. Open-ended questions invited participants to further expand on their thoughts (e.g., “Write two recommendations that you believe would improve UASC referral to your mental health service”). Three online surveys, adapted to the expertise of each group of participants were constructed and piloted in a convenience sample to identify any problems before the implementation of the survey. Table 1 depicts the mapping topics that were addressed.

To assess how facility coordinators and field psychologists perceived their work culture, we selected 14 items from the Organizational Culture Profile (OCP) [20] that reflected humanitarian values, such as fairness, risk taking, innovation, and stability, rather than business-related values. Respondents were asked to rate how well each of the listed values described their organization, using a 9-point scale, ranging from “not at all” (1) to “a great extent” (10). The internal consistency of the 14-item scale measured with Cronbach’s alpha coefficient was 0.936.

### 2.4. Quantitative Analyses

Descriptive statistics were used to conduct quantitative analyses, using the MS Excel and Statistical Package for Social Sciences (SPSS 28.0). To test for differences between groups, the chi-square test was used for categorical data, whereas for mean group differences with respect to continuous data, *t*-tests were used, with a set level of significance of 0.05.

### 2.5. Qualitative Analyses

The qualitative data involved an analysis of open-ended questions, inviting either a specification (e.g., “Which are the psychosocial difficulties that UASC encounter?”) or an argumentation (e.g., “How psychosocial services can be improved?”). Even though the responses are descriptive, they generate rich and spontaneous information. Popping [21] suggests that the coding of open-ended responses is performed either from an instrumental perspective (answers are interpreted according to the researcher’s theory) or from a representational perspective (answers reflect the participants’ perspective). We adopted the latter, and carried out a qualitative content analysis [22,23] for the few open-ended questions that were included in the survey. The advantage of this method is that information is directly obtained from participants without imposing preconceived categories and allowing their experience to emerge. To reduce the risk of bias, three researchers read all answers to the open-ended questions, identified codes, which were grouped into categories and sub-categories.

## 3. Results

Guided by the five research questions of the mapping approach, findings are presented below in five sections:

### 3.1. Profile of UASC and Field Workers Supporting Them

#### 3.1.1. Profile of UASC Living in Long-Term Accommodation Facilities

Based on the data provided by the facility coordinators, 798 UASC were accommodated in shelters and SILs when the study was carried out. The vast majority were boys older than 14 years of age (78.3%), whereas few (11.2%) were under 13 years of age. Girls older than 14 years (7.3%) or younger than 13 years (3.3%) were significantly fewer. They originated from 21 different countries, with Afghanistan (39.7%), Pakistan (18.4%), and Syria (11.4%) being the most common countries of origin. The remaining countries included Iraq, Iran, Somalia, Bangladesh, Myanmar, Guinea, Eritrea, Kuwait, Egypt, Algeria, Morocco, Cameroun, Ghana, Gambia, Kongo, Kurdistan, Palestine, and Sierra Leone. These data reflect the composition of UASC in Greece during the time of the study, in terms of age range, gender, and country of origin [17].

Regarding their legal status, a relatively small number were granted either refugee status (*n* = 98; 12.3%) or a stay permit for humanitarian reasons (*n* = 8; 1%). Most were either awaiting a decision on their asylum application (55.5%) or a decision for family reunification or re-location to another European country (26.4%), and 4.8% were denied asylum in Greece.

The duration of residing in the facility varied, with most having lived in the same facility for either 6–12 months (43%), or less than 6 months (38.3%). A small proportion (10.1%) were accommodated for up to 18 months and an even smaller proportion (8.6%) for up to 24 months or more.

Only one facility coordinator responded that none of the hosted UASC were enrolled in formal education. According to the remaining 33 facility coordinators, a total of 468 (58.8%) UASC were enrolled in formal education, and only few (3.6%) in vocational training. Almost half of facility coordinators (47.1%) reported adolescents’ unwillingness to enroll in or regularly attend school despite efforts and actions made in this direction. The reasons for not attending school were diverse. These included: lack of afternoon preparatory classes (55.9%), the absence of an intercultural school near the location of residence (26.5%), change of accommodation facility in the middle of the school year (35.3%), collaboration difficulties with the school principal (26.5%), bullying incidents by peers, and negative reactions by the school parents’ associations (20%).

#### 3.1.2. Profile of Field Workers Providing Psychosocial Support to UASC

According to facility coordinators’ data provision, overall, 632 field workers were responsible for the care of 798 UASC living within the survey sites. The largest group of employees comprised caregivers (30.1%), followed by interpreters (11.4%), psychologists (10.3%), social workers (10.3%), educators (8.7%), and lawyers (5.5%). The auxiliary personnel included cleaners (4.9%), catering staff (5.7%), and security staff (9.7%).

The vast majority of employees across all professional groups and types of accommodation held short-term contracts, e.g., 91.9% of the full-time psychologists, 86.2% of the full-time social workers, 86.2% of part-time educators, and 84.8% of the full-time caregivers.

Examining the average staff–child ratio, this was higher in shelters than in SIL apartments across all professional groups. Concerning the psychologists, the average ratio was 12 and 23 UASC per psychologist in shelters and in SIL apartments, respectively. This ratio was corroborated by the information provided by the 28 field psychologists who were assigned to meet the psychosocial needs of 491 UASC; 46.4% of field psychologists reported providing support to 11–20 young people at the same time; 28.6% to less than 10 UASC; and 25% to more than 21 young people.

Legal assistance during the asylum application process was available on a regular basis for UASC living in most shelters and in all SILs; in 2 out of 33 shelters (6%), it was available on an hoc basis, two to four days per month.

Regarding interpreters, the situation varied. In residential structures, 14.7% of facility coordinators reported a complete absence of interpreters, while 11.7% experienced varying availability, ranging from 6 to 15 days per month. The majority were speakers of Farsi and Arabic. In the absence of an interpreter, assistance was sought from minors who could communicate in either English or Greek. Specifically, 206 (25.8%) UASC were reported to be able to communicate in Greek with staff members.

### 3.2. Psychosocial and Mental Health Needs of UASC

#### 3.2.1. UASC’s Psychosocial Challenges as Perceived by Field Psychologists and CAMHS Directors

**Figure 2:**
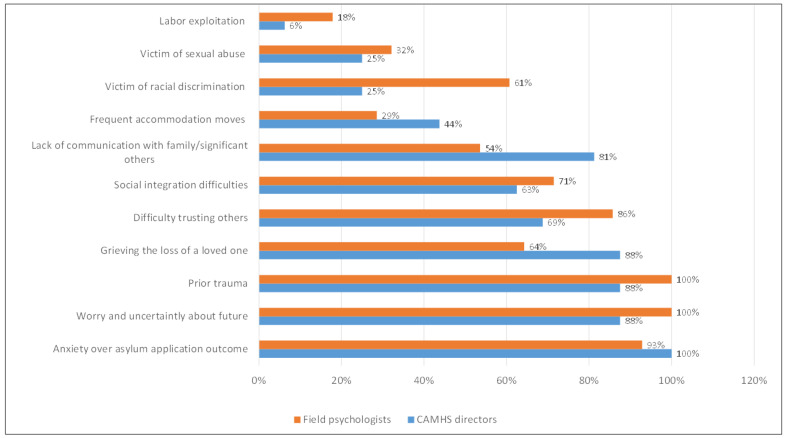
CAMHS directors and field psychologists perceived psychosocial challenges faced by UASC.

The most common distressing lived experiences identified by field psychologists and CAMHS directors were related to UASC anxieties about their asylum application outcomes, worry and uncertainty about the future, and trauma-related issues. Grieving the loss of a loved one and struggling with trusting others were, respectively, the second and the third most commonly reported perceived challenges by both groups of professionals (Figure 2). Psychologists were notably more likely than CAMHS directors to perceive UASC as victims of racial discrimination (60.7% versus 25% respectively, *χ*^2^ = 5.21, *p* = 0.023).

#### 3.2.2. UASC’s Psychosocial Needs as Perceived by Field Psychologists

The thematic analysis of responses to the open-ended question “What are the UASC’s two most important psychosocial needs?” highlighted the need for establishing relationships of trust, a sense of security and safety, and social and emotional support. Some pointed out the need for acceptance and social/school integration. The analysis of responses as to what, in their opinion, would UASC identify as their most important needs, participants highlighted: (a) the need for support in safeguarding the legal process of asylum applications, and (b) the need for acceptance, safety, and social inclusion. In terms of what psychologists perceived as the minors’ desires about their future, an overwhelming majority (91.4%) reported the wish to work and earn money, and, for about two thirds (66.9%), the wish to move to another European country.

#### 3.2.3. UASC’s Mental Health Needs as Perceived by Field Psychologists

In terms of specific distressing emotions, thoughts, and behavior problems displayed by UASC, field psychologists were asked to report the frequency with which these occurred. The results indicated that sleeping difficulties/nightmares, somatic symptoms (e.g., headaches), worry and uncertainty about the future, were reported to occur often by over 50% of the field psychologists. Crying, sadness, intense anxiety, irritability, mood liability, self-harm, withdrawal, reduced engagement in play or other activities, expressions of intense nostalgia, thoughts of worthlessness, and violation of residence rules were reported as occurring sometimes by over 40% of psychologists. Conduct type problems, such as substance or alcohol use, aggressive/violent behavior towards others outside the residential setting, behaviors that put their life or the lives of others at risk, and abusive behavior against staff members were reported as never or rarely occurring by over 40% of field psychologists.

The thematic analysis of responses to the open-ended question “What are the two UASC mental health problems that are most challenging to the staff” indicated aggression and behavioral problems, followed by self-harm and suicidal ideation.

Regarding UASC mental health problems, 58.7% (469/798) were reported to display behavioral and/or emotional disturbance. A third (34%, 271/798) were referred for a psychiatric assessment: 12.4% (99/798) to public community-based CAMHS, 8.4% (67/798) to hospital-based CAMHS, 3.9% (31/798) to private psychiatrists, 7.9% (63/798) to mental health services operated by NGOs, and 1.4% (11/798) to a specialized program for substance use problems. Among those referred, 27.7% (75/271) required inpatient psychiatric care, of whom 41.3% (31/75) were compulsorily admitted to the hospital. All UASC who exhibited psychotic symptoms or attempted suicide were promptly referred for psychiatric assessment. For other mental health issues, the referral rate varied (Table 2). Among those who received outpatient psychiatric care, approximately one-third (31.3%) attended 1–3 sessions, half (50%) 4–6 sessions, and about a fifth (18.8%) attended more than 7 sessions; only 12.5% of the CAMHS services offered a follow-up appointment, whereas the remaining only did so in cases where it was needed or requested. In the view of field psychologists, 16% of the referred UASC showed ‘great’ improvement, 40% ‘some’ improvement, 36% ‘slight’ improvement, and 8% ‘no change’ or a ‘worsening’ of behavioral and emotional difficulties.

### 3.3. MHPSS Services Provided to UASC

#### 3.3.1. Range of Psychosocial Services Provided in Accommodation Settings

In terms of how the mental health difficulties of UASC are addressed by the personnel in residential settings, field psychologists reported the following five approaches as commonly employed: (a) offering psychological support from an on-site psychologist, providing emotional support and strategies to manage psychological distress (96.4%); (b) pursuing a referral to a community mental health specialist for assessment and treatment (78.6%); (c) attending staff meetings to discuss the challenges faced by UASC, aiming at facilitating problem-solving and creating a supportive environment (78.6%); (d) attending meetings held by the personnel with all the UASC of the same residential settings to share and address common issues (71.4%); and (e) developing or updating individualized care management plans for addressing mental health needs (71.4%).

#### 3.3.2. Range of Mental Health Services Provided by CAMHS

**Figure 3:**
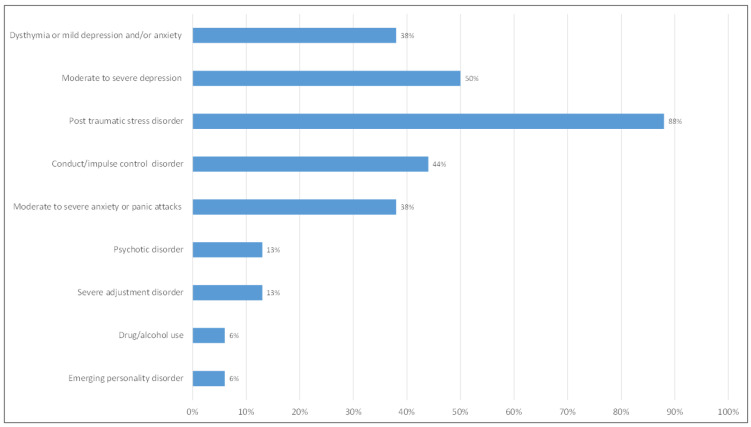
Diagnoses made by CAMHS with regards to referred UASC.

According to CAMHS directors, the three most frequent reasons for referral to CAMHS included violent/aggressive behavior, self-harm behaviors, depression symptoms, and/or suicidal ideation. A total number of 729 UASC (77.5% boys) attended hospital-based (67.2%) or community-based (32.8%) CAMHS over a 2-year period (2018–2019), of whom 42.1% (307/729) received an emergency psychiatric assessment with a view to potential involuntary admission following a legal order issued by the Prosecutor of Minors, triggered by the request of shelter staff; of those, 77.6% (239/307) were assessed as not requiring admission. Overall, 12.5% (91/729) of UASC referred to CAMHS required inpatient psychiatric care (voluntary 3% and involuntary 9.5%). The inspection of the graphical representation of the data regarding the frequency of diagnoses (Figure 3) reveals that the three most commonly reported were PTSD, moderate to severe depression, and conduct/impulse control disorder. Severe/moderate anxiety disorder or panic attacks, and dysthymia or mild anxiety and/or depression disorder, were less prevalent.

Regarding inpatient care, its duration ranged from less than a week (14.3%), one to four weeks (27.6%), up to a two-month period (57.1%), throughout which collaboration with the staff at the shelters was maintained with regards to drawing up a care plan after discharge. Just above half (57%) of hospitalized minors returned to their living accommodation; for a substantial proportion (43%), the admission was extended because the shelters refused to take them back and there was a delay in finding a new placement.

Concerning mental health care provision, all the directors of CAMHS indicated pharmacological management and brief individual counseling. A little over half (56.3%) reported providing supervision, once to twice per month, to staff in shelters. In case of follow-up, collaboration was maintained with the key worker (56.3%), the facility coordinator (18.8%), or the minor’s legal guardian (18.8%). Written feedback with recommendations was provided by just under half of CAMHS (43.4%), usually in the case of hospitalized minors. A vast majority (93.6%) reported a lack of mechanisms to monitor and evaluate the effectiveness of treatment interventions.

### 3.4. Challenges to Providing Effective MHPSS Services to UASC

#### 3.4.1. Referral and Collaboration Issues Among Field and CAMHS Personnel

According to field psychologists, one of the significant challenges related to timely assessments and interventions for UASC with mental health issues is the prolonged wait time for specialist appointments after referral. The average wait time for an appointment in the public sector was 20 ± 9 days for hospital-based CAMHS (min 7, max 30) and 40 ± 33 days for community-based CAMHS (min 7, max 120); for the mental health service operated by NGOs, this was 49 ± 47 days (min 5, max 120), and in private sector, this was 5 ± 3 days (min 2, max 7). Additional obstacles related to accessing CAMHS included the long distance between the shelter’s location and CAMHS site or lack of available service in the area (60.7%).

A significantly higher proportion of CAMHS directors, compared to field psychologists, reported incomplete information concerning the minors’ social and mental health history as an obstacle to psychiatric evaluation, *χ*^2^ = 12.09, *p* = 0.001 (75% vs. 21.4%, respectively), and inefficient coordination between services (mental health, school, police, and residential setting), *χ*^2^ = 6.04, *p* = 0.014 (62.5% vs. 25%, respectively).

Both field psychologists and CAMHS directors identified inefficient communication with the minor due to a lack of, or an inadequacy in, interpretation (64.3% vs. 75%); difficulties responding to additional health needs (e.g., ordering tests or prescribing medication), due to the lack of a social security number (60.7% vs. 43.8%); non-prioritization of UASC’s needs (32.1% vs. 12.5%); and ineffective collaboration between CAMHS and facility staff (28.6% vs. 25%). Meanwhile, racist/discriminatory behavior against UASC by CAMHS staff was reported by a minority (10.7% vs. 12.5%, respectively).

Additional obstacles in providing care, reported only by CAMHS directors, included understaffing (43.8%), absence of the child’s key worker during the appointment (56.3%), concerns about accuracy of information provided by the minor (68.8%), staff lacking cultural competence and expertise in working with this population (25%), a lack of therapeutic residential units to accommodate UASC with severe mental health problems (43.8%), unnecessarily prolonged hospitalization due to a difficulty in finding a new accommodation placement for UASC (25%), the UASC’s nonattendance to scheduled appointments (18.8%), and the UASC’s non-adherence to prescribed medication (6.3%).

#### 3.4.2. Challenges Regarding Provision of Psychosocial Support in Shelters

The following challenges were identified by field psychologists with respect to the provision of psychosocial support services to UASC: (a) communication issues with UASC, due to the absence or unavailability of cultural mediators/interpreters (71.4%); (b) team issues due to the lack of a shared philosophy of care among staff members (53.6%); communication problems and conflicts among team members (32.1%); a lack of supervision (53.6%) and insufficient staff training (46.4%); and (c) organizational issues, caused by understaffing (32.1%), uncertainty about one’s job position (32.1%), non-prioritization of UASC psychosocial support needs (32.1%), and a lack of funds for activities aiming at building the youngsters’ resilience (60.1%).

#### 3.4.3. Perceived Work Culture in Shelters

A significant difference was found between field psychologists and facility coordinators in their overall perception of work culture in the organization’s daily operations, *t*(60, 40.514) = 3.108, *p <* 0.01 (88.25 ± 21.28 and 102.26 ± 11.90, respectively). The examination of the individual items reflecting distinct characteristics of organizational culture revealed that field psychologists as compared to facility coordinators rated significantly lower the following values: innovation (*p* = 0.001), risk-taking (*p* = 0.006), experimenting (*p* = 0.006), opportunities (*p* = 0.007), and stability (*p* = 0.004). No significant differences were found on items referring to tolerance, rule-orientation, and collaboration.

### 3.5. Recommendations by Survey Participants

#### 3.5.1. Team Issues

Field psychologists suggested several measures to overcome the aforementioned challenges and enhance psychosocial support for UASC. Their top priorities included clarifying the roles and responsibilities of team members, implementing a shared care plan to boost the psychosocial health of UASC, and ensuring the presence of cultural mediators. When questioned about what they believe UASC would suggest if asked about what would enhance their psychosocial support, the majority of psychologists indicated a more enriched daily life with activities both within and outside the facility.

#### 3.5.2. Ongoing Training and Staff Supervision

**Figure 4:**
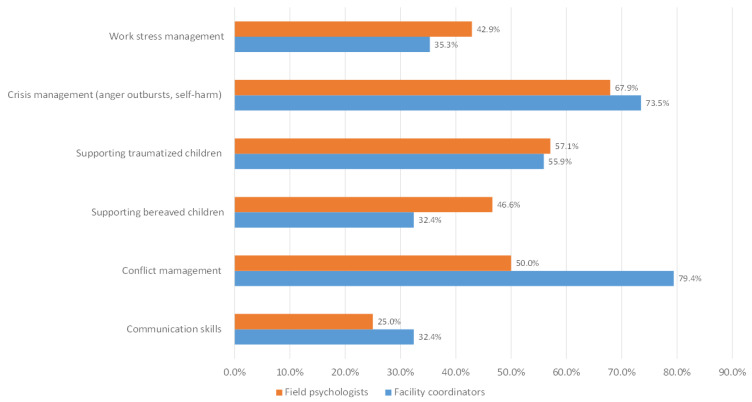
Perceived training needs by facility coordinators and field psychologists.

The need for ongoing in-house training and staff supervision was underscored. When compared to psychologists, facility coordinators seemed to place a higher emphasis on the necessity of training in the areas of crisis management, conflict management, and communication skills; conversely, they seemed to place a lower emphasis on work stress management and supporting bereaved children (Figure 4).

#### 3.5.3. Improvement of Referral Pathways

The majority of respondents from both groups recognized enhancing the referral process as a necessity. Directors of CAMHS expressed the need for consistent collaboration and communication with psychosocial care providers at the facilities. Many emphasized the importance of having specialized mental health staff with proper training in residential settings and having sufficient information about the minors’ life history. Conversely, field psychologists pointed out the need for written feedback, follow-ups by CAMHS, and the provision of psychotherapy by a psychologist who is external to the facility.

## 4. Discussion

Following the 2015–2016 refugee crisis, Greece has been compelled to create an emergency system to secure the care and protection needs of unaccompanied minors. In this context, the path to safe long-term accommodation in shelters or SILs was established, offering protection from precarious living conditions, and providing minors with access to legal advice, to school education, to health services, and opportunities for social integration. Although numerous conventions and EU directives transposed into national legislation impose certain standards and secure access to health and MHPSS services, the actual situation has been reported to be very different in practice [24]. In this study, we explored the perceptions of facility coordinators, field psychologists, and community mental health professionals regarding the psychosocial and mental health needs of UASC residing in long-term accommodation facilities. We also examined how these needs are addressed, when, by whom, and with which resources. To our knowledge, this is the first study of its kind in Greece, a country most often perceived by UASC a steppingstone in the pursuit of their goals to settle in a prosperous region of northern Europe.

### 4.1. Perceived UASC’s Psychosocial and Mental Health Needs

In terms of UASC’s psychosocial needs, establishing relationships of trust that enhance a sense of safety and security, social and emotional support are the most commonly identified priority needs by the survey respondents. School enrolment is identified as a major challenge, given that only a very small minority are enrolled in vocational training, and several minors are unwilling to enroll in or regularly attend school. High mobility in this population (e.g., 38% of UASC lived in the same facility for less than 6 months), and the ill-prepared Greek education system to receive refugees (e.g., a limited number of afternoon preparatory classes teaching basic language skills or schools not always welcoming refugees) contribute to the low school enrollment/attendance rates.

According to field psychologists, a significant majority of unaccompanied asylum-seeking children (UASC) express a desire to work and earn money; two-thirds wish to move to another European country, with just over half hoping to do so through family reunification schemes. Meanwhile, the vast majority are awaiting decision on their asylum applications. The lengthy asylum processes in Greece can lead to UASC feeling a loss of control over their lives, which heightens their uncertainty about the future while they concurrently cope with past trauma-related experiences. Psychological distress manifestations, including sleeping difficulties/nightmares, sadness, irritability, mood liability, headaches, intense nostalgia, reduced engagement in play or other activities, and thoughts that nobody cares for them have been identified as common, whereas aggression and disruptive behavior problems, followed by self-harm and suicidal ideation are perceived as the most challenging behaviors for facility caregivers. Our findings indicate that within a span of one year, nearly 59% of UASC in the under-study facilities showed symptoms of emotional or behavioral difficulties. More than half of these minors were recommended for psychiatric evaluation. These results align with other studies that have explored mental health challenges among unaccompanied refugee minors [7,9,25,26,27]. It is noteworthy that among those who were assessed to require inpatient psychiatric care, more than a third were involuntarily admitted to the hospital. It is important to note that over 90% of cases that were referred due to self-harm, violent behavior, depression, or alcohol/substance use did not result in hospitalization. Concomitantly, the data provided by the CAMHS directors indicates a relatively high rate (42%) of emergency psychiatric evaluation with a view to potential involuntary admission, initiated by a legal order from the Prosecutor of Minors, which was triggered by a request from the staff at the shelter. Interestingly, more than three quarters (77%) of UASC who were compulsorily referred were evaluated as not requiring hospitalization.

In total, 12.5% of the UASC evaluated by the CAMHS in the study required inpatient psychiatric care. This included 3% who were voluntarily admitted and 9.5% who were involuntarily admitted. These figures are higher than the reported 3% of involuntary admissions for the local adolescent population, as noted in the study by Voultsos et al. (2020). These findings align with another European study that emphasizes that UASC are more likely to require inpatient psychiatric care compared to the native population [28].

Taken together, these findings may reflect the inexperience and confidence of the staff at the facilities managing mental health challenges of UASC. They also highlight the barriers to accessing mental health services as a result of substantial shortfalls in the Greek public health system, i.e., a relatively weak primary health care in terms of access, care integration, and continuity in care [29], which are circumvented through the Prosecutor of Minors’ order for compulsory admission. Other studies have found that access to mental health services relies on whether social service agencies or guardians recognize the minors’ mental health needs [30], and that only the most severe presentations are likely to be brought to the attention of mental health services [31,32]. Interestingly, some studies highlight a marked overrepresentation of UASCs in both voluntary and involuntary inpatient psychiatric care [28].

### 4.2. The Nature of Services, Gaps, Obstacles, and Good Practices in Supporting UASC

The overwhelming majority of employees who work in long-term facilities hold short term contracts, thus plausibly evoking job insecurity and rendering the task of continuity of care hard to accomplish. Although at first glance, the average staff–child ratio in shelters appears satisfactory, this is not the case for those living in SILs. Concomitantly, most respondents report limited training in supporting young people in distress, with prior loss and traumatic experiences, and in managing conflict and mental health/behavior crisis. Within this context and given the lack of clearly defined needs-led care pathways in the community, it is not surprising that extremely challenging behaviors (e.g., self-harming, violent behavior, and severe anger outbursts) displayed by UASC often lead to crisis-driven responses through public tertiary health services, following the Prosecutor of Minors’ order for a compulsory psychiatric admission upon the request of shelters’ personnel when the case cannot be handled within the accommodation facility.

An alarming finding that merits attention is the unnecessary extension of hospital stays in nearly half of the cases due to the challenges in securing a new placement for the young person following their discharge. Additionally, our findings suggest that in the context of out-patient care, there may be an underuse of psychological services in the UASC population. Both hospital- and community-based CAMHS offer pharmacological treatment and counseling, whereas brief trauma-informed psychotherapy seems to be available only to a limited number of young people. The absence of a mechanism to monitor and assess the effectiveness of both inpatient and outpatient interventions raises serious concerns about whether the mental health needs of unaccompanied minors, who come to the attention of CAMHS, are adequately met.

Turning to the nature of the psychosocial support provided on-site, language barriers are identified as strongly hindering individual and group psychological interventions with UASC of different ethnic background, given that the availability of interpreters varies across facilities. An additional factor that influences the type of services provided is the perceived work culture. Interestingly, field psychologists rated the overall organizational culture significantly lower than facility coordinators did. This was particularly true for values such as innovation, risk-taking, experimenting, opportunities, and stability—all of which contribute to personal and professional growth. These findings may reflect a case-management approach for UASC that follows a predetermined by-the-book approach, leaving little room for alternative or systemic strategies. In such a milieu, the absence of continuous training and staff supervision deprives field workers of a shared philosophy and approach to supporting UASC and managing highly challenging behaviors, such as violent outbursts, self-harm, and suicidal threats.

Respondents identified several additional challenges, including issues with accessibility due to long distances from CAMHS or a lack of available service in the area, the absence of clear referral and care pathways, ineffective collaboration between CAMHS and shelter personnel, a lack of established mechanisms for monitoring mental health outcomes in the course of psychological or/and pharmacological interventions. Undoubtedly, all the above factors hinder timely assessment and intervention, which can lead to a mental and behavioral deterioration, negatively affecting the caregivers at the facilities and impacting on daily life within the residential setting.

Overall, the above findings highlight the difficulties in providing psychosocial support to UASC in Greece. These challenges are perpetuated by the limitations of a public care system that has been overburdened by years of austerity measures and is unable to meet the mental health needs of children and adolescents in the country. The situation is further exacerbated by professionals who are ill-prepared in providing care to minors with diverse cultural backgrounds. Similar findings have been reported by researchers who compared child protection frameworks in Greece, Italy, and Spain [12]. This comparative study highlighted how the child protection frameworks in these three countries can retraumatize minors, due to fragmented, discontinuous, and frequent changes in the living environment (e.g., high staff turn-over) and malpractices that lead to social exclusion and even the exploitation of minors.

Despite the identified gaps and challenges in MHPSS service delivery, several good practices in the psychosocial care of UASC have been identified. These include: (a) the provision of a variety of structured activities, such as leisure, sport, and creative and cultural group activities prior to the COVID-19 pandemic-related restrictions by two thirds of the shelters; (b) the facilitation of exchange and dialogue through meetings held between staff and all UASC living in the same residential setting, reported by more than two-thirds of the facilities; and (c) the focus on emotional wellbeing through the provision of individual psychological support on-site by field psychologists when needed.

### 4.3. Limitations and Further Research

The results of this study should be viewed and interpreted in consideration of its limitations. Firstly, a mapping survey does not allow us to validate the information provided by participants, nor does it allow us to gather data about the quality of the services provided. The fact that we did not ask the UASC themselves about their essential needs and obstacles in accessing MPHSS services is a limitation that should be addressed in future study. Secondly, the varied participation rates among facility coordinators, field psychologists, and CAMHS directors, coupled with the lack of input from other professionals who provide care to UASC (e.g., social workers, educators, etc.) limit the generalizability of our findings. Thirdly, the present data is insufficient to draw conclusions about the impact of living “betwixt and between” countries on shaping the UASC’s lived experience and its effect on their short-term and long-term wellbeing. Previous studies involving UASC have demonstrated that mental health difficulties may be linked not only to traumatic events experienced before and during their journey, but also to stressors encountered after arriving in the host country, where they face an uncertain legal status and an unpredictable future [33,34].

A notable strength of this study is that the findings reflect the experience of a sample that comprise facility coordinators and field psychologists from all NGOs bar one that provide long-term accommodation for UASC and mental health professionals in CAMHS, located in various cities, towns, and islands across Greece. Our results provide valuable insights for researchers, project managers, and policymakers in Greece to devise strategies that effectively address the identified gaps and challenges in MHPSS provision for UASC, thereby helping to alleviate the mental health burden they experience. While the findings may not be generalizable to UASC residing in other types of settings, such as camps, our mapping approach may prove useful in other refugee contexts for identifying the minors’ mental health needs, tailoring MHPSS services to meet them, and empowering with resources the professionals who assume their care.

## 5. Conclusions

The findings of the present study have broader implications for future research and the development of MHPSS services for UASC residing in long-term accommodations. They highlight: first, the need to explore how UASC perceive the country in which they are temporarily hosted or wish to settle in, given that their perceptions and aspirations can influence their commitment to a structured life, their desire for social integration, and their ability to cope with the stress of the asylum process; second, the need to prevent lengthy delays in asylum application procedures, which can exacerbate existing mental health problems; third, the need to develop a comprehensive, sustainable plan that offers opportunities for learning the local language, gaining an education, and receiving vocational training, which can help minimize or prevent chronic distress, illegal flight to other countries, and future labor exploitation; fourth, the implementation of a robust, coordinated, and periodically evaluated strategic national plan to support UASC with psychosocial and mental health issues, through establishing effective referral pathways to facilitate service coordination among stakeholders; and, lastly, the need for regular supervision and ongoing training on intercultural mental health issues for both facility and community professionals supporting UASC. The establishment of practice guidelines that promote a transcultural approach and the implementation of structured training programs are equally vital. These initiatives will benefit service designers, MHPSS providers, schools, and local communities seeking to integrate unaccompanied minors and learn from their experiences.

## Figures and Tables

**Table 1:** 5-W mapping topics.

		Survey 1. Facility Coordinators	Survey 2. Field Psychologists	Survey 3. CAMHS Directors
1.	WHO?	Facility personnel	Field psychologists	Mental health professionals
2.	WHERE?	Geographical location	Geographical location (Similar to that of facility coordinators)	Geographical location
3.	WHAT?	UASC’s psychosocial needs and living conditions	UASC’s psychosocial, mental health needs, and the nature of psychosocial support	UASC’s mental health symptoms/disorders and nature of service provision
4.	WHEN?	Referral procedures and collaboration with CAMHS	Referral procedures and collaboration with CAMHS	Collaboration with the personnel of facility accommodation
5.	WHICH?	Resources, gaps, obstacles, work culture, and recommendations	Resources, gaps, obstacles, work culture, and recommendations	Resources, gaps, obstacles, and recommendations

**Table 2:** Mental health symptoms, referrals to CAMHS, and hospitalization during 2019.

		Referred to CAMHS	Voluntary Admission	Compulsory Admission
Type of symptoms	N (%)	N (% within type of symptoms)	N (% within type of symptoms)	N (% within type of symptoms)
Psychotic symptoms	28 (6)	28 (100)	8 (28.6)	7 (25)
PTSD symptoms	71 (15.1)	49 (69)	5 (7)	3 (4)
Suicide attempt	14 (3)	14 (100)	8 (57.1)	6 (42.9)
Depression symptoms	83 (17.7)	49 (59)	5 (6)	3 (3.7)
Anxiety/Panic attacks	45 (9.6)	28 (62.2)	4 (8.9)	1 (2.2)
Self-harm behavior	61 (13)	35 (57.4)	2 (3.3)	3 (4.9)
Violent behavior	79 (16.8)	34 (43%)	3 (3.8)	3 (3.8)
Alcohol/Substance use	87 (18.6)	33 (37.9)	3 (3.4)	3 (3.4)
Other	1 (0.2)	1 (100)	0 (0)	0 (0)
TOTAL	469 (100)	271 (57.8%) ^a^	44 (16.2%) ^b^	31 (11.4%) ^b^

^a^ calculated as number of referred/total number presenting with any type of symptoms ^b^ calculated as number of involuntary admitted/number of referred.

## Data Availability

The original contributions presented in this study are included in this article. Further inquiries can be directed to the corresponding author.
